# Bloomingdale Asylum for the Insane

**Published:** 1845-03

**Authors:** 


BLOOMINGDALE ASYLUM FOR THE INSANE.

To Dr. Pliny Earle, physician to the institution, we are indebted for an able report on the present state of the asylum for the insane, at Bloomingdale, in New York. The report is very full, and embraces a multitude of details of the highest interest. Of 106 patients in the hospital, 50 were cured in the course of the year, and 6 others are reported as being detained until their cure, now far advanced, shall be confirmed. It is plain that this institution is not behind any other in our country in the adoption of those improved modes of managing the insane, which are among the boasts of modern practical medicine. Y.

